# Editorial Changes

**Published:** 1880-01

**Authors:** 


EDITORIAL CHANGES.—Those who notice the external title page of this number of the JOURNAL AND EXAMINER, will see that the name of William H. Byford, A.M., M.D., has disappeared from the list of the corps of editors. When the *Chicago Medical Journal* and the *Medical Examiner*, were united and placed in the hands of the Chicago Medical Press Association, the directors of that organization selected Prof. Byford as the senior editor for a term of four years. He had completed his full term of service last month, and declined a re-appointment. His association with his colleagues during the entire four years has been pleasant and profitable. While they will miss his valuable counsel in the direction of their work, we trust the readers of the JOURNAL AND EXAMINER will continue to be favored with the perusal in its pages of such contributions as his time and ripe experience will enable him to make. D.

